# Tomato

**DOI:** 10.1093/jxb/eraf395

**Published:** 2025-11-25

**Authors:** Yves Gibon, Diane M Beckles, Sonia Osorio, Hiroshi Ezura

**Affiliations:** INRAE, Univ. Bordeaux, UMR 1332, Biologie du Fruit et Pathologie, 33882 Villenave d’Ornon, France; Department of Plant Sciences, University of California, Davis, CA 95616, USA; The Institute for Mediterranean and Subtropical Horticulture ‘La Mayora’, Department of Molecular Biology and Biochemistry, University of Malaga-Spanish National Research Council (IHSM-UMA-CSIC), 29010 Malaga, Spain; Institute of Life and Environmental Sciences, University of Tsukuba, 1-1-1 Tennodai, Tsukuba, Ibaraki 305-8572, Japan

**Keywords:** Abiotic stress, biotic stress, fruit, genomics, light, metabolism, metabolomics, phytohormones, tomato, transcription factors

## Abstract

Tomato is both one of the most consumed horticultural crops and the model species for fleshy fruits. While impressive yields are now obtained, the tomato sector must face the numerous threats to these advances in productivity due to the consequences of climate change. Since the first draft genome sequence in 2012, our understanding of tomato genetics has advanced dramatically, greatly facilitating the identification of mechanisms involved in growth, development, resistance to stress, and organoleptic quality. This intensive investigation has broadened our understanding of the integration and coordination of these mechanisms by phytohormones and transcription factors, as well as the roles played by metabolism, particularly in establishing fruit quality, with the aim to better target breeding programmes and to develop innovative cultivation practices.

## Superlative vegetable crop

Tomato (*Solanum lycopersicum*) is one of humanity’s favourite vegetables. Its annual production continues to grow, reaching nearly 200 Mt in 2023 worldwide (80% for the fresh market and 20% for processing) for a gross production value of more than US$100 billion (https://www.fao.org). This represents ∼25 kg per year per inhabitant, which in particular meets a significant portion of the daily recommended amount of vitamin C (75 mg per day; a medium-sized tomato, ∼100 g, contains between 10 mg and 20 mg). However, tomato provides many other healthful nutrients including antioxidants such as lycopene, other carotenoids and polyphenols, as well as amino acids and fibre. Tomato is a climacteric fruit, which allows harvesting before maturity, offering flexibility in its supply chain management. Whether in the field or in a greenhouse, the plant is relatively easy to cultivate, and it is often profitable for farmers. Thus, this species of subtropical South American origin, today cultivated on five continents and at many latitudes, has become a cornerstone of global agriculture.

## Tomato challenges

Stabilizing and increasing yield, which reaches up to 75 t ha^–1^ in the field and up to 600 t ha^–1^ in high-tech greenhouses, remains a major challenge for the sector. Yield results from the complex regulation of source–sink relationships and the avenues for managing it are varied, whether it involves maximizing photosynthesis per unit of cultivated area, optimizing the distribution of trusses relative to that of leaves, or controlling the appearance of suckers. There is often a trade-off between yield and nutritional quality, which results from the composition of minerals and metabolites beneficial to health. Similarly, there is a trade-off between shelf-life and organoleptic or nutritional quality. Better understanding of the mechanisms involved in these trade-offs will certainly be important, because quality, both health and sensory, is increasingly sought after by consumers.

Tomato cultivation is threatened by numerous abiotic and biotic stresses. Greenhouses generally offer protection from adverse environments, but heat stress, connected with climate change is problematic. Above 32 °C, it causes male sterility and can promote blossom-end rot when mineral nutrition is inadequate. In the field, also affected by heat waves, the frequency and intensity of drought and flooding are increasing with climate destabilization. Among emerging biotic threats, the tomato brown rugose fruit virus (ToBRFV) represents perhaps the most pressing challenge facing the industry, spreading rapidly worldwide since its 2014 emergence and defying current control strategies. Transmitted by seeds and by simple contact, it is currently a very serious threat to the sector, as no treatment or resistance has been found so far (Zhang *et al*., 2022).

The tomato breeding industry is flourishing, with an estimated annual turnover of ∼US$1 billion that continues to grow yearly. It benefits in particular from the existence of closely related species with which *S. lycorpersicum* can be sexually crossed and which are sources of potentially useful alleles for improving yield, nutritional quality, and stress resistance (Moreels *et al*., 2023). Tomato is also easy to transform, which enables genome editing in addition to classical breeding and genomic selection.

Optimizing crop management is a further major challenge for the tomato industry, but smart solutions, as demonstrated for example by the very high yields obtained by injecting CO_2_ into greenhouses, can be devised. However, to guarantee stable yields, radical solutions will have to be found to mitigate the effects of increasing climate instability, such as high-tech innovations, shifting the growing season, and relocation, but also breeding more robust varieties.

## Tomato research

Tomato has long been the model species for fleshy fruits. It is easy to cultivate, has a relatively short reproductive cycle (10–14 weeks for the miniature Micro-Tom variety), and well-characterized phenotypic diversity. It is a diploid with a modest sized genome (950 Mb) that was first sequenced in 2012 (The Tomato Genome Consortium, 2012). Many improvements have since been made to its assembly, and the number of accessions and related species sequenced continues to grow. Numerous genetic resources are available, such as collections of intra- and interspecific diversity or recombinant populations resulting from crosses with related species (Li *et al*., 2023). This diversity has been widely exploited to identify quantitative trait loci (QTLs) for traits of interest and even to dissect the underlying genes and mechanisms. Mutant collections have enabled interesting phenotypes and their causal genes to be identified, notably through mapping-by-sequencing. The great ease of transformation of this self-pollinating species facilitates rapid candidate gene testing for functional genomics and crop improvement.

## In this issue

This Special Issue features seven reviews (including a Darwin review), one expert view, 12 research articles, and two technical innovations, all focused on the major challenges facing the tomato industry.

Our robust understanding of the tomato genome structure provides a valuable foundation for tomato research and improvement. The review by Rose *et al*. (2025) shows how far genomics has progressed in tomato and its related species. The orchestration of gene expression through the joint action of phytohormones and transcription factors is a major topic in tomato research. The Darwin review dissects the involvement of four hormones (abscisic acid, ethylene, jasmonate, and salicylate) in stress responses and in fruit development and ripening (Jaryal *et al*., 2025), and four research papers address various aspects of hormone action and metabolism (Castro-Estrada *et al*., 2025; Nguyen *et al*., 2025; Nomura *et al*., 2025; Pardo-Hernandez *et al*., 2025). The integration of transcriptomics and metabolomics performed by Pardo-Hernandez *et al*. (2025) exemplifies the power of multiomics strategies for dissecting complex stress responses. The kinetic model linking ethylene biosynthesis to signalling proposed by Nguyen *et al*. (2025) represents a significant methodological advance towards the prediction of fruit ripening. The review by Wang and de Maagd (2025) and two research papers (Jo *et al*., 2025; Martínez-Estrada *et al*., 2025) update the involvement of transcription factors in various processes, including fruit development and ripening, root suberin formation, and fruit nutritional quality. Metabolism also plays a major role in yield, resistance to stress, and the development of fruit quality (Bertin *et al*., 2025; Rodriguez-Concepcion *et al*., 2025). In just over 25 years, metabolomics has become an essential tool for fruit research and could prove useful for breeding (Karakas and Fernie, 2025).

Hybridization is often preferred by breeders because it eases the introduction of complex and multigenic traits. Thus, the relevance of cytoplasmic male sterility to avoid the long and costly process of inbreeding is discussed by Iki *et al*. (2025). In this context, a technical innovation is proposed to isolate male gametophytes at high throughput (Flores-Tornero *et al*., 2025). Another way to exploit genetic diversity is grafting with other *Solanum* species, which gives promising results in terms of yield (Ganie *et al*., 2025).

Tomato cultivation practices benefit from innovations resulting from a better understanding of its physiology. Thus, Heuvelink *et al*. (2025) discuss the effects of light regimes on tomato performance as well as the mechanisms involved. The use of biostimulants, whether chemicals, microorganisms such as mycorrhizae, or composts, is another possible avenue for improving cultivation practices (Giovannini *et al*., 2025; W. Li *et al*., 2025). A highly original application of mechanical treatments to improve yield is proposed by Castro-Estrada *et al*. (2025).

Tomato has low tolerance to stress. Two articles address hypoxia, a constraint that has still been relatively little studied in tomato (Eerdekens *et al*., 2025; X. Li *et al*., 2025). Root suberization, involved in response to stress, particularly water stress, is studied using a functional genomics approach (Jo *et al*., 2025). Finally, two articles deal with biotic stress: the role of trichomes in defence against insects is studied by Popowski *et al*. (2025) and a technical innovation for the rapid detection of viruses, including ToBRFV, is proposed (Hak *et al*., 2025).

## Conclusion

An analysis of the literature indicates an exponential increase in the number of studies on tomato over the past 10 years. The topics covered in this Special Issue are fully representative of this enthusiasm for tomato. They represent a good match between the growing economic importance and the ease of experimentation that characterize this species. Much effort was devoted to elucidating mechanisms, particularly gene functions. Integrating this knowledge in relation to the new challenges facing the sector, in particular climate change, reduced pesticide use, and improved nutritional quality, will undoubtedly require the addition of further approaches such as modelling and, more generally, artificial intelligence to the arsenal already deployed to better understand the biology of tomato and that of fruits in general, and to control their production. This may in particular allow a better understanding of the influence of the microbiota, a question which adds further complexity but will undoubtedly become essential in agriculture of the future.
